# Rethinking gonadotropin releasing hormone agonist suppression test: why it should be a gonadotropin releasing hormone antagonist suppression test

**DOI:** 10.1210/jendso/bvag191

**Published:** 2026-09-11

**Authors:** Rayyan Munshi, Louis-Philippe Coutu Nadeau, Michael H Dahan

**Affiliations:** Division of Reproductive Endocrinology and Infertility, McGill University Health Centre, Montreal, QC, Canada H2L 4S8; Division of Reproductive Endocrinology and Infertility, McGill University Health Centre, Montreal, QC, Canada H2L 4S8; Division of Reproductive Endocrinology and Infertility, McGill University Health Centre, Montreal, QC, Canada H2L 4S8

**Keywords:** GnRH agonist, GnRH antagonist, diagnostic suppression testing, hyperandrogenism, reproductive endocrinology

## Abstract

The gonadotropin-releasing hormone (GnRH) suppression test has traditionally used GnRH agonists to differentiate gonadotropin-dependent from gonadotropin-independent sex steroid production. Although physiologically sound, this approach is limited by an initial luteinizing hormone and follicle-stimulating hormone (FSH) flare and the prolonged interval required to achieve pituitary suppression. We revisit the physiologic basis of the traditional GnRH agonist suppression test and propose the hypothesis that GnRH antagonists may provide a more direct pharmacologic approach to diagnostic suppression testing. Unlike GnRH agonists, antagonists produce immediate competitive receptor blockade without an initial stimulatory flare, resulting in rapid suppression of pituitary gonadotropins. However, downstream testosterone and estradiol concentrations are expected to decline more gradually because of hormone synthesis and physiologic clearance. This modification, in theory, should be more efficient for suppression testing. However, the optimal timing of downstream hormone reassessment remains unknown and requires prospective clinical investigation. Antagonist-based suppression testing should therefore be regarded as a hypothesis-generating concept rather than an established clinical strategy. If prospectively validated, it may provide a practical refinement of conventional GnRH agonist-based suppression testing while preserving its physiologic foundation.

The gonadotropin-releasing hormone (GnRH) suppression test is used to determine whether sex steroid production is driven by pituitary gonadotropins or by an autonomous source. Traditionally, this test has been performed using long-acting GnRH agonists, such as leuprolide acetate, or repeated administration of shorter-acting agonist preparations. The underlying physiologic principle is straightforward: if suppression of pituitary luteinizing hormone (LH) and follicle-stimulating hormone (FSH) results in a corresponding reduction in downstream testosterone or estradiol concentrations, hormone production is considered gonadotropin dependent. Conversely, persistent elevation of sex steroid concentrations despite suppression of pituitary gonadotropins suggests autonomous hormone production (1, 2).

Although this diagnostic approach is rarely used in routine clinical practice, it remains a well-established physiologic test and continues to be described in endocrinology and reproductive medicine references. The physiologic rationale underlying the GnRH agonist suppression test remains sound, and the test has historically provided a useful method for distinguishing gonadotropin-dependent from gonadotropin-independent hormone production in selected clinical situations (1).

However, the continued use of GnRH agonists for diagnostic suppression testing warrants reconsideration in light of advances in GnRH pharmacology. Unlike GnRH agonists, which require an initial stimulatory phase before receptor desensitization and suppression are achieved, GnRH antagonists produce immediate competitive blockade of pituitary GnRH receptors and rapidly suppress gonadotropin secretion without an initial flare. These pharmacologic differences raise the question of whether the same physiologic principle underlying the traditional suppression test could be achieved more efficiently using a GnRH antagonist (2, 3).

Importantly, the availability of a pharmacologically attractive alternative does not diminish the physiologic validity of the traditional GnRH agonist suppression test. Rather, it provides an opportunity to reconsider whether the diagnostic objective of suppression testing could be achieved through a more direct and theoretically rapid mechanism. At present, however, this concept has not been evaluated, and its diagnostic performance relative to conventional GnRH agonist-based suppression testing remains unknown.

Against this background, we propose the hypothesis that GnRH antagonist-based suppression testing may provide a practical alternative to the traditional GnRH agonist suppression test while preserving its underlying physiologic principles. The pharmacologic rationale supporting this hypothesis, together with its potential advantages, limitations, and future research requirements, is discussed below.

## Physiologic basis of the traditional GnRH agonist suppression test

The traditional GnRH agonist suppression test is based on a well-established physiologic principle of pituitary regulation. Continuous exposure to a GnRH agonist initially stimulates pituitary GnRH receptors, producing a transient increase in LH and FSH secretion. With continued nonpulsatile receptor stimulation, however, pituitary GnRH receptors become desensitized and downregulated, resulting in suppression of gonadotropin secretion. If downstream testosterone or estradiol concentrations subsequently decline, hormone production is considered gonadotropin dependent. In contrast, persistent elevation of sex steroid concentrations despite gonadotropin suppression suggests autonomous hormone production (1, 2).

This physiologic principle remains valid and has historically provided the foundation for diagnostic suppression testing. The principal limitations of the traditional approach are therefore not related to its biologic rationale, but rather to the pharmacologic characteristics of GnRH agonists required to achieve pituitary suppression (1, 2).

## The initial flare response

Administration of a GnRH agonist produces an initial stimulatory phase before pituitary receptor desensitization occurs. During this flare response, transient increases in LH and FSH secretion result in temporary elevations of downstream sex steroid production. Although this response represents a normal pharmacologic consequence of GnRH agonist administration and does not invalidate the diagnostic test, it contributes little to the diagnostic objective itself. Rather than facilitating interpretation, the transient increase in gonadotropin and sex steroid concentrations may complicate the clinical assessment of hormonally active conditions and delay achievement of the desired suppressed endocrine state.

Importantly, the flare response should therefore be viewed as a pharmacologic characteristic of GnRH agonists rather than a necessary component of diagnostic suppression testing (2).

## Time required for pituitary suppression

A second practical limitation of GnRH agonist-based suppression testing is the time required to achieve adequate pituitary suppression. Because receptor downregulation develops gradually after continuous nonpulsatile receptor stimulation, diagnostic reassessment frequently requires approximately 2 weeks, depending on the agonist formulation and dosing regimen. Although this interval reflects the normal pharmacodynamics of GnRH agonists, it prolongs the diagnostic process and may delay clinical decision-making (1, 2).

These practical considerations do not diminish the physiologic validity of the traditional GnRH agonist suppression test. Instead, they raise the question of whether the same diagnostic objective could be achieved more efficiently using an alternative pharmacologic strategy that suppresses pituitary gonadotropin secretion without an initial stimulatory phase.

## Potential role of GnRH antagonists

Gonadotropin-releasing hormone antagonists provide rapid competitive blockade of pituitary GnRH receptors without the initial stimulatory flare characteristic of GnRH agonists (3, 4).

From a physiologic perspective, the diagnostic objective of suppression testing remains unchanged regardless of the pharmacologic agent employed. In both approaches, the fundamental question is whether suppression of pituitary gonadotropins results in a corresponding reduction in downstream testosterone or estradiol production. Thus, the rationale for considering GnRH antagonists is not based on a different diagnostic principle, but rather on the possibility of achieving the same physiologic endpoint through a more direct pharmacologic mechanism.

Previous pharmacodynamic studies have demonstrated that injectable GnRH antagonists swiftly suppress pituitary gonadotropin secretion, with substantial reductions in LH occurring within approximately 4 to 8 hours after administration, whereas FSH suppression develops over the subsequent 24 hours. These observations suggest that pituitary suppression can be achieved considerably earlier than with conventional GnRH agonist-based protocols (4, 5).

Importantly, rapid suppression of LH does not necessarily result in an immediate decline in downstream testosterone or estradiol concentrations. Gonadal steroid concentrations reflect not only pituitary gonadotropin secretion but also ongoing hormone synthesis, secretion, distribution, metabolism, and physiologic clearance. Consequently, suppression of downstream sex steroid concentrations is expected to lag slightly behind the initial decline in LH and FSH.

**Figure 1:**
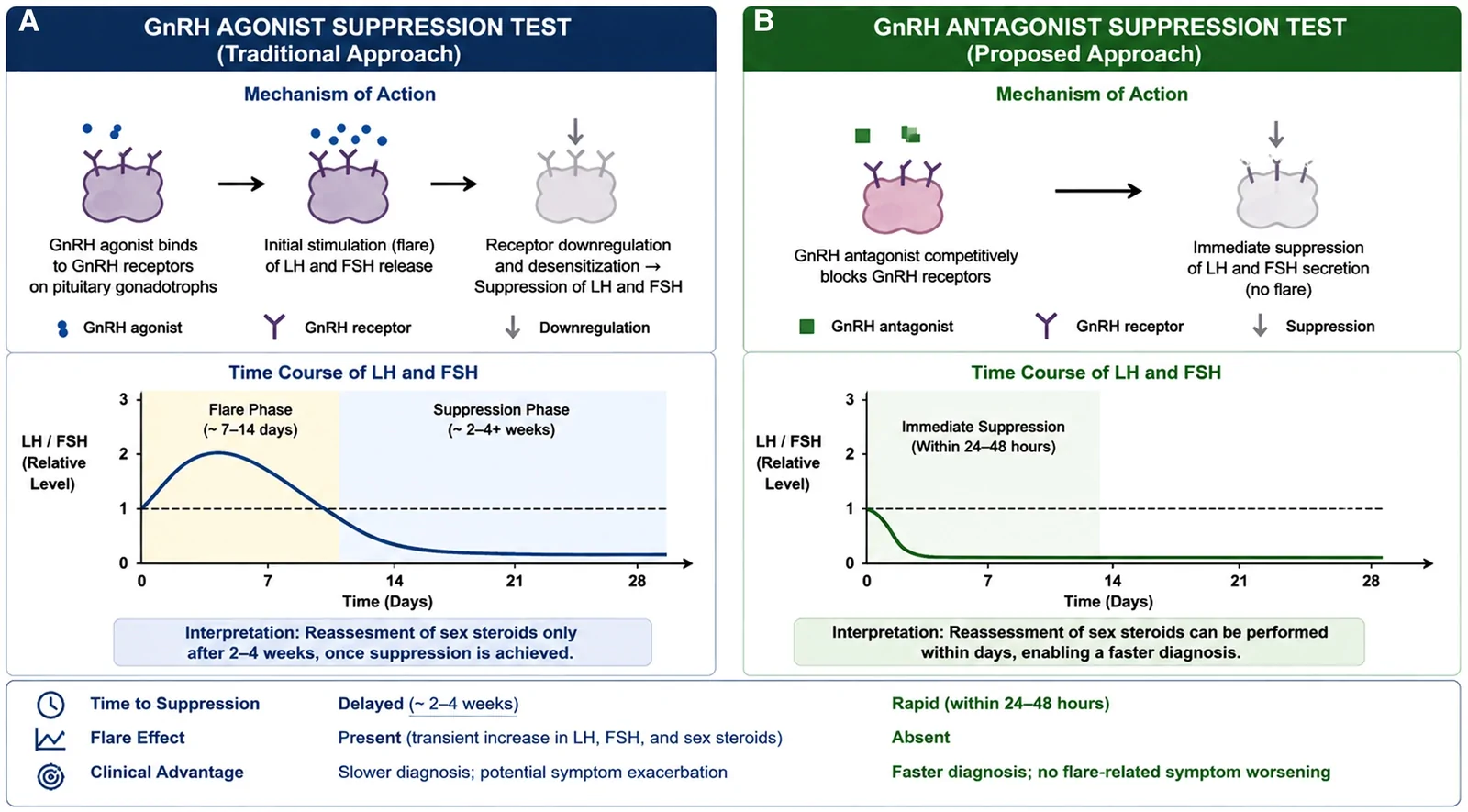
Proposed comparison of GnRH agonist- and GnRH antagonist-based suppression testing. Gonadotropin-releasing hormone agonists initially stimulate pituitary GnRH receptors, producing a transient flare in luteinizing hormone (LH) and follicle-stimulating hormone (FSH) secretion before receptor desensitization and gonadotropin suppression occur. In contrast, GnRH antagonists produce rapid competitive receptor blockade without an initial flare, with substantial LH suppression occurring within approximately 4 to 8 hours and FSH suppression developing over the subsequent 24 hours. Downstream testosterone and estradiol suppression is expected to follow pituitary gonadotropin suppression and may become evident over approximately 24 hours. The antagonist-based diagnostic pathway shown is conceptual and requires prospective clinical validation (2-7).

This concept is supported by published pharmacodynamic studies. In healthy men receiving the GnRH antagonist cetrorelix, maximal suppression of testosterone occurred several hours after the initial decline in LH, demonstrating that downstream steroid suppression follows rather than coincides with pituitary gonadotropin suppression (5). Similarly, studies of the oral GnRH antagonist elagolix in premenopausal women demonstrated rapid suppression of gonadotropins, while suppression of estradiol became evident over the subsequent 24 hours (6, 7). Collectively, these findings suggest that although GnRH antagonists rapidly suppress pituitary function, the optimal timing for reassessment of downstream sex steroid concentrations remains uncertain and should be established through prospective clinical investigation. Although, based on prior studies, this would be expected to occur in about 24 hours (5-7) (Fig. 1).

Accordingly, the potential advantages of GnRH antagonists should not be interpreted as evidence of immediate clinical superiority over traditional GnRH agonist-based suppression testing. Rather, they provide a pharmacologically plausible rationale for evaluating whether the same diagnostic objective can be achieved more efficiently while preserving the physiologic principles underlying suppression testing.

## Oral GnRH antagonists

The availability of orally administered GnRH antagonists raises the possibility of further simplifying diagnostic suppression testing. Agents such as elagolix and relugolix provide rapid competitive blockade of pituitary GnRH receptors and have well-characterized pharmacokinetic and pharmacodynamic profiles (6-8). Their oral administration may improve patient convenience and facilitate outpatient suppression testing.

However, their application in diagnostic suppression testing remains theoretical. Although oral GnRH antagonists rapidly suppress pituitary gonadotropins, their diagnostic performance, optimal dosing, and timing of downstream hormone reassessment have not been established. Prospective studies are therefore required before oral formulations can be considered for clinical suppression testing.

## Limitations and future directions

Although the pharmacologic rationale for antagonist-based suppression testing is compelling, several important questions must be addressed before this concept can be translated into routine clinical practice.

First, the proposed approach has not been validated, and no comparative clinical studies have evaluated the diagnostic performance of GnRH antagonist-based suppression testing relative to the traditional GnRH agonist suppression test. Consequently, its diagnostic accuracy, sensitivity, specificity, and reproducibility remain unknown. However, in theory, GnRH antagonist suppression testing should work.

Second, the optimal timing for reassessment of downstream sex steroid concentrations following GnRH antagonist administration has not been established. Available pharmacodynamic studies demonstrate rapid suppression of pituitary gonadotropins; however, testosterone and estradiol decline over a longer interval because of the kinetics of hormone synthesis and physiologic clearance. Prospective studies are therefore required to determine the most appropriate timing of hormone measurement for diagnostic suppression testing. However, it should be noted that in studies, suppression usually occurs within 24 hours of taking the GnRH antagonist, in terms of estradiol and testosterone production (5-7).

Third, standardized diagnostic thresholds have not been established for antagonist-based suppression testing. Future investigations will need to define appropriate biochemical cutoffs and determine whether these differ from those used in traditional GnRH agonist-based protocols.

Practical considerations, including medication availability, cost, route of administration, and patient selection, should also be evaluated before widespread clinical implementation can be considered.

Future validation of this hypothesis should ideally be undertaken through prospective comparative clinical studies. Standardized antagonist-based suppression protocols should be compared directly with conventional GnRH agonist suppression testing to evaluate diagnostic concordance, optimal timing of hormone measurements, safety, patient convenience, and overall clinical utility. Establishing standardized protocols and diagnostic thresholds will be essential before antagonist-based suppression testing can be considered for routine clinical practice.

## Conclusion

The traditional GnRH agonist suppression test remains a physiologically sound diagnostic approach for distinguishing gonadotropin-dependent from gonadotropin-independent sex steroid production. Nevertheless, its reliance on an initial stimulatory flare and the prolonged interval required for pituitary receptor desensitization highlight practical limitations that reflect the pharmacologic characteristics of GnRH agonists rather than shortcomings of the underlying physiologic principle.

Gonadotropin-releasing hormone antagonists provide a rapid competitive receptor blockade without an initial flare and swiftly suppress pituitary gonadotropin secretion (3-7). These pharmacologic properties provide a compelling rationale for investigating whether antagonist-based suppression testing can achieve the same diagnostic objective more efficiently than conventional GnRH agonist-based protocols. However, available evidence remains limited to pharmacodynamic observations, and the diagnostic performance of antagonist-based suppression testing has not been evaluated.

Accordingly, antagonist-based suppression testing should currently be regarded as a hypothesis-generating concept rather than an established clinical diagnostic strategy. Prospective comparative studies are required to determine its diagnostic accuracy, establish standardized testing protocols, define optimal timing for downstream hormone assessment, and evaluate its overall clinical utility.

If validated, GnRH antagonist-based suppression testing may provide a practical refinement of the traditional GnRH agonist suppression test while preserving its physiologic foundation.

## Data Availability

No new data were generated or analyzed in support of this commentary.
